# Dopamine, cognitive function, and gamma oscillations: role of D4 receptors

**DOI:** 10.3389/fncel.2013.00102

**Published:** 2013-07-02

**Authors:** Katrina E. Furth, Surjeet Mastwal, Kuan H. Wang, Andres Buonanno, Detlef Vullhorst

**Affiliations:** ^1^Section on Molecular Neurobiology, Eunice Kennedy Shriver National Institute of Child Health and Human Development, National Institutes of HealthBethesda, MD, USA; ^2^Graduate Program for Neuroscience, Boston UniversityBoston, MA, USA; ^3^Unit on Neural Circuits and Adaptive Behaviors, Genes, Cognition, and Psychosis Program, National Institute of Mental HealthBethesda, MD, USA

**Keywords:** dopamine, D4 receptor, gamma oscillations, parvalbumin, fast-spiking interneurons, prefrontal cortex, schizophrenia, ADHD

## Abstract

Cognitive deficits in individuals with schizophrenia (SCZ) are considered core symptoms of this disorder, and can manifest at the prodromal stage. Antipsychotics ameliorate positive symptoms but only modestly improve cognitive symptoms. The lack of treatments that improve cognitive abilities currently represents a major obstacle in developing more effective therapeutic strategies for this debilitating disorder. While D4 receptor (D4R)-specific antagonists are ineffective in the treatment of positive symptoms, animal studies suggest that D4R drugs can improve cognitive deficits. Moreover, recent work from our group suggests that D4Rs synergize with the neuregulin/ErbB4 signaling pathway, genetically identified as risk factors for SCZ, in parvalbumin (PV)-expressing interneurons to modulate gamma oscillations. These high-frequency network oscillations correlate with attention and increase during cognitive tasks in healthy subjects, and this correlation is attenuated in affected individuals. This finding, along with other observations indicating impaired GABAergic function, has led to the idea that abnormal neural activity in the prefrontal cortex (PFC) in individuals with SCZ reflects a perturbation in the balance of excitation and inhibition. Here we review the current state of knowledge of D4R functions in the PFC and hippocampus, two major brain areas implicated in SCZ. Special emphasis is given to studies focusing on the potential role of D4Rs in modulating GABAergic transmission and to an emerging concept of a close synergistic relationship between dopamine/D4R and neuregulin/ErbB4 signaling pathways that tunes the activity of PV interneurons to regulate gamma frequency network oscillations and potentially cognitive processes.

## INTRODUCTION

Schizophrenia (SCZ) is characterized by three distinct symptom clusters – positive, negative, and cognitive. Historically, positive symptoms such as hallucinations and delusions have received the most attention, and are often improved by typical and atypical antipsychotics. By contrast, negative symptoms such as social withdrawal, lack of motivation, impaired social interactions, and cognitive impairments including deficits in attention, working memory, and executive function are refractory to antipsychotics. The intractability of cognitive symptoms has been a major obstacle in the development of more effective therapies for SCZ, especially in light of epidemiological findings indicating that the severity of cognitive symptoms is more predictive of long-term prognosis than positive symptoms (Green, 1996), and that cognitive impairments are already present at the prodromal stage (Reichenberg et al., 2002).

Cognitive impairments may arise from altered activity in the prefrontal cortex (PFC). Imaging studies suggest hypofunction of the PFC and reduced signal-to-noise ratios during cognitive tasks in schizophrenic patients (Winterer and Weinberger, 2004; Tost et al., 2005). Local oscillatory network activity in cortical areas, in particular gamma-band rhythms (30–80 Hz), correlate with working memory and selective attention (Womelsdorf and Fries, 2007; Benchenane et al., 2011), and higher cognitive demands correlate with increased gamma power and coherence across different cortical areas (Cho et al., 2006; Basar-Eroglu et al., 2007). Numerous studies have shown that basal, evoked, and induced gamma oscillations are altered in SCZ patients (Herrmann and Demiralp, 2005; Uhlhaas and Singer, 2010). Most of these studies focused on evoked oscillations in sensory cortices, in particular the auditory cortex for its prominent role in auditory hallucinations. Patients with SCZ fail to increase gamma oscillation power in the PFC in response to increased cognitive demands, suggesting reduced signal-to-noise (Cho et al., 2006). Gamma oscillations are an inherent property of recurrent networks of interneurons and pyramidal neurons, and arise by the synchronization of pyramidal neuron output by fast-spiking (FS) basket cell and chandelier interneurons expressing the Ca^2^^+^ binding protein parvalbumin (PV). The central role of PV-expressing interneurons for the generation of gamma rhythms has recently been demonstrated using optogenetic techniques *in vivo* (Cardin et al., 2009; Sohal et al., 2009). Moreover, numerous postmortem studies in persons with SCZ have shown changes in PV interneurons, in particular decreases in mRNA and protein expression of GAD67 (glutamic acid decarboxylase 67 kD) and PV itself (Lewis et al., 2011), consistent with the notion of perturbed maturation of inhibitory PFC circuits during adolescence (Benes and Berretta, 2001; Beneyto and Lewis, 2011; Sullivan and O’Donnell, 2012).

It was initially thought that the dopamine (DA) system was generally overactive in SCZ because DA D2-type receptor antagonizing antipsychotic medications mitigated many observed symptoms. However, this original DA hypothesis has evolved over the years to account for evidence from imaging, genetic and metabolic imaging studies more consistent with hypoactivity of frontal cortical circuits in SCZ, hypoactivity of the mesoprefrontal DA system and hyperactivity of mesostriatal and mesonigral DA systems (reviewed in Simpson et al., 2010). While the importance of dopaminergic modulation of the PFC for proper cognitive functions is well supported by experimental evidence from non-human primates and rodents (Goldman-Rakic, 1995; Robbins and Arnsten, 2009), how altered DA activity contributes to PFC hypofunction in SCZ is not understood. Moreover, given its implication in attention and working memory, surprisingly little is known about the possible role of DA signaling in the regulation of oscillatory activity in the PFC and hippocampus, another major allocortical structure implicated in SCZ (Harrison, 2004).

Here we review the current state of research into the role of DA in the modulation of network rhythms in the cortex, particularly the PFC and hippocampus, as new evidence emerges that DA signaling is potently involved in regulating theta and gamma oscillations. A main focus of the review is the D4 receptor (D4R), considered a major D2-type receptor in the forebrain and prominently expressed in PV-expressing interneurons. Recent studies have shown that DA signaling via D4R, in a close and synergistic relationship with the neuregulin 1 (NRG-1)/ErbB4 signaling pathway, itself genetically implicated in SCZ, modulates gamma rhythms in the hippocampus (Andersson et al., 2012b). Taken together with the genetic association of the 7-repeat (7R) variant of the human *DRD4* gene with increased risk for attention deficit hyperactivity disorder (ADHD) and altered gamma-band responses (Demiralp et al., 2007; Gizer et al., 2009), these findings should renew interest in the D4R and its possible contribution to synchronization of PFC networks during cognitive tasks.

## FAST-SPIKING INTERNEURONS AND GAMMA OSCILLATIONS

Gamma oscillations are generated through recurrent networks of FS interneurons interconnected by chemical and electrical synapses (for reviews, see Bartos et al., 2007; Colgin, 2011; Whittington et al., 2011; Buzsaki and Wang, 2012). Most FS interneurons are PV-expressing basket cells that form perisomatic connections with multiple pyramidal neurons, faithfully release GABA (gamma-aminobutyric acid) in response to depolarization (Hefft and Jonas, 2005), and discharge action potentials on almost every gamma cycle (Gloveli et al., 2005). Together, these properties make them perfectly suited to entrain pyramidal neurons. In fact, using *in vivo* optogenetic stimulation, depolarizing PV-expressing interneurons increased gamma power, whereas hyperpolarizing PV-expressing interneurons decreased gamma power (Sohal et al., 2009). Moreover, work in the somatosensory cortex suggests that gamma oscillations can be more effectively induced *in vivo* by 40-Hz optogenetic stimulation of PV-expressing interneurons than of pyramidal neurons (Cardin et al., 2009), although optogenetic depolarization of pyramidal neurons has been shown to generate gamma oscillations *in vivo* in the somatosensory cortex (Adesnik and Scanziani, 2010) and in slices from the medial PFC (Yizhar et al., 2011) as well. Notably, optogenetic depolarization of both PV-positive and pyramidal neurons generate gamma synchrony even when stimulated non-rhythmically, supporting the notion that gamma oscillations are an emergent property of coupled networks of excitatory and inhibitory cells (Cardin et al., 2009; Yizhar et al., 2011; for a review, see Tiesinga and Sejnowski, 2009).

### EXCITATION/INHIBITION BALANCE

Proper balance of excitatory and inhibitory transmission onto PV interneurons is essential for the generation of normal gamma rhythms. Hippocampal slices prepared from mice with PV interneuron-selective ablation of the GluA1 subunit of the AMPA (alpha-amino-3-hydroxy-5-methyl-4-isoxazole propionic acid) receptor have reduced power of kainate-induced gamma oscillations in area CA3 (Fuchs et al., 2007). PV interneuron-selective ablation of the GluN1 subunit of the NMDA (*N*-methyl-D-aspartate) receptor resulted in elevated spontaneous gamma rhythms in the hippocampus of awake behaving mice (Korotkova et al., 2010), but impaired gamma rhythm induction in the somatosensory cortex in response to optogenetic stimulation (Carlen et al., 2012). Surprisingly, mice lacking GABAergic transmission onto PV interneurons by selective ablation of δ and γ2 subunits of the GABA_A_ receptor have normal gamma oscillations but severe disruptions in theta oscillations and theta-gamma coupling, suggesting that mutual inhibition of PV interneurons is dispensable for *in vivo* gamma oscillations (Wulff et al., 2009).

*N*-methyl-D-aspartate receptors on FS interneurons have long been considered critical for normal excitation/inhibition balance in cortical microcircuits and are at the center of the glutamate hypofunction theory of SCZ (Lisman et al., 2008; Nakazawa et al., 2012). This hypothesis is based on findings that dissociative anesthetics that antagonize NMDA receptors such as ketamine and phencyclidine (PCP) elicit psychotic symptoms in normal individuals similar to those found in SCZ subjects and exacerbate psychosis in patients (Kantrowitz and Javitt, 2012). As discussed above, compelling evidence from gene targeting studies implicates NMDA receptors in gamma synchrony. However, a recent study comparing postnatal vs. adult ablation of the GluN1 subunit suggests that NMDA receptors on FS interneurons might be more important for the proper maturation of cortical circuits and the emergence of gamma oscillation during development than for a direct contribution to synaptic processes underlying gamma oscillation induction or propagation (Belforte et al., 2010; Korotkova et al., 2010). Consistent with this idea, electrophysiological studies in wild-type mice show that excitatory postsynaptic potentials (EPSPs) on FS interneurons have large contributions from NMDA receptors in juveniles that diminish during maturation (Wang and Gao, 2009; Rotaru et al., 2011). Notably, since NMDA receptors have slower kinetics than AMPA receptors, the summating properties of excitatory synapses with a large NMDA receptor contribution could make them less suitable to support gamma rhythms. Therefore, excitatory drive is dominated by AMPA receptors in mature FS interneurons to ensure high temporal precision between excitation and spiking (Rotaru et al., 2011).

### PHENOTYPIC ALTERATIONS OF PV INTERNEURONS IN SCZ

Postmortem studies have consistently revealed decreased levels of GAD67 mRNA in the dorsolateral PFC (DLPFC) and hippocampus of SCZ subjects, particularly in PV interneurons (for reviews, see Benes and Berretta, 2001; Gonzalez-Burgos and Lewis, 2012). As GAD67 synthesizes the inhibitory neurotransmitter GABA, it seems reasonable to speculate that diminished GAD67 expression reduces inhibitory drive, perturbs normal excitation/inhibition balance, and thereby impairs neural synchrony. Interestingly, SCZ subjects also have reduced PV expression in the cortex (Hashimoto et al., 2008), a finding that has been suggested to represent a compensatory response to lower GAD67 levels, as PV-deficient mice exhibit increased GABA release and kainate-induced hippocampal gamma oscillations power and frequency (Vreugdenhil et al., 2003). An alternative interpretation, based on the marked dependence of the expression of both GAD67 and PV genes on neural activity, is that reduced levels of GAD67 and PV are a consequence of abnormally low activity of FS interneurons in SCZ subjects.

Work in rodents suggests that GAD67-mediated GABA synthesis has an additional role in shaping GABAergic innervation of cortical networks during adolescence, a period in which PV interneuron networks mature and gamma oscillations become prevalent (Chattopadhyaya et al., 2007). Reduced GAD67 expression during adolescence could therefore conceivably cause persistent changes in GABAergic innervation, resulting in perturbed excitation/inhibition balance and dysfunctional cortical networks in affected individuals. Lastly, it has been proposed that because of their high metabolic demand, FS interneurons are particularly vulnerable to oxidative stress during development. Consistent with this notion, studies have identified abnormal antioxidant systems in SCZ subjects, and work in rodents has demonstrated that exposure to oxidative stress causes loss of PV-immunoreactivity in FS interneurons and reductions in high-frequency network oscillations (Behrens and Sejnowski, 2009; Do et al., 2009).

## GAMMA OSCILLATIONS IN SCZ

While abnormalities in neural oscillations have been identified in essentially all frequency bands in patients with SCZ (Moran and Hong, 2011), gamma-band activity is particularly affected and might represent an endophenotypic biomarker of its etiology (Uhlhaas and Singer, 2012). This notion is based on reports of high heritability of altered auditory and visual steady-state evoked potentials (SSEPs) in affected individuals and their first-degree relatives (for example, Hall et al., 2011), and on studies finding that perturbations in gamma-band responses present early in the course of the disorder and are independent of medication status (Gallinat et al., 2004; Symond et al., 2005; Haenschel et al., 2009; Minzenberg et al., 2010).

In general, high-frequency beta and gamma oscillations synchronize local networks, while low-frequency delta, theta, and alpha oscillations establish synchronization over longer distances (von Stein and Sarnthein, 2000). Similarly, gamma oscillations between the PFC and cortical sensory areas mediate attention by enhancing the neuronal representation of attended sensory input, while theta oscillations between the PFC and hippocampus mediate working and long-term memory (Benchenane et al., 2011). A given neuron may participate in several of these frequencies at any given time, and measures of cross-frequency coupling indicate how well one frequency is embedded in another. Evidence of abnormal neural oscillations in SCZ comes from a large number of clinical studies primarily using electroencephalography (EEG) and magnetoencephalography (MEG). These studies revealed changes in sensory-evoked gamma oscillations, oscillations that correlate with propensity for hallucinations, and abnormal modulation of neural oscillations during cognitive tasks.

In “bottom-up” approaches, patients have been shown to have trouble generating, maintaining, and propagating SSEPs entrained by stimuli presented at gamma-band frequency, with impairments in both phase-locking and amplitude. Similarly, “top-down” perceptual processing paradigms such as auditory oddball tasks, sustained tones, speech sounds, and button-press tasks mostly revealed impairments in evoked gamma power (Haig et al., 2000) and synchrony (for an excellent review on both approaches, see Uhlhaas and Singer, 2010). Lastly, evoked oscillations triggered by direct transcranial magnetic stimulation are markedly reduced in patients with SCZ and are confined to the stimulated brain region, whereas gamma oscillations propagate to neighboring cortical regions in healthy controls (Ferrarelli et al., 2008).

Apart from lower stimuli-evoked gamma oscillations, affected individuals also show reduced high-frequency activity at rest (Rutter et al., 2009). Interestingly, they also have a greater number of uncorrelated gamma oscillations at rest, especially between the PFC and other cortical areas, resulting in reduced coordination of cortical activity (Yeragani et al., 2006; Salvador et al., 2010; Kikuchi et al., 2011). The variety of concurrent but uncorrelated oscillations in various areas could be described as “noise” because they potentially mask salient signals evoked from sensory cortices. Gamma oscillation signal-to-noise ratios are reduced in SCZ during cortical information processing, due to elevated spontaneous activity and reduced evoked responses (for example, Winterer et al., 2000; reviewed in Gandal et al., 2012).

Task demands with a strong cognitive component have also been used to reveal gamma deficits in SCZ patients. For example, patients with SCZ have abnormal oscillations during lexical encoding (Xu et al., 2013), and do not display typical increases in gamma activity during mental arithmetic (Kissler et al., 2000). During working memory tasks, healthy controls increase gamma oscillations in frontal cortical areas as the task demands increase. However, SCZ patients show maximal gamma oscillatory activity for simple working memory tasks as well as difficult ones (Cho et al., 2006; Basar-Eroglu et al., 2007). Moreover, gamma power during encoding and maintenance positively correlates with performance in these tasks, and SCZ patients show deficits in producing this delay-period gamma oscillation (Cho et al., 2006; Haenschel et al., 2009; Minzenberg et al., 2010).

Several studies show that increased high-frequency oscillations in primary sensory cortices correlate with the propensity for auditory hallucinations in SCZ patients, even when subjects are not actively hallucinating (Baldeweg et al., 1998; Lee et al., 2006; Mulert et al., 2011). An increased tendency for neural circuits to enter states of high-frequency synchronization has been suggested to reflect hyperexcitability in sensory cortices (Spencer et al., 2009).

## DOPAMINE’S ROLE IN NEURAL OSCILLATIONS AND COGNITION

The importance of DA neurotransmission for attention and normal cognitive function has long been recognized. Deficiencies in mesoprefrontal dopaminergic circuits in humans are associated with major psychiatric disorders such as SCZ, ADHD, and addiction (Winterer and Weinberger, 2004; del Campo et al., 2011; Goldstein and Volkow, 2011). In rodents and non-human primates, DA modulates PFC neuron activity during cognitive tasks and motivational behaviors (Goldman-Rakic, 1995; Grace et al., 2007; Robbins and Arnsten, 2009), and pharmacological lesions of dopaminergic projections to the PFC greatly impair working memory (Brozoski et al., 1979). DA effects on cognitive performance exhibit an inverted U-shaped dose–response curve (Sawaguchi and Goldman-Rakic, 1994; Vijayraghavan et al., 2007). In cortical areas, extracellular DA is cleared mostly by enzymatic inactivation catalyzed by the product of the catechol-*O*-methyltransferase gene (*COMT*). A functional variation exists in the human population (Val158Met) in which the Val allele renders the enzyme significantly more active than the Met allele, thereby inactivating extracellular DA more rapidly. Homozygous carriers of the Val allele are therefore believed to have lower prefrontal DA levels and indeed show reduced performance in working memory and executive function tasks (Goldberg et al., 2003). Moreover, amphetamine improves prefrontal signal-to-noise ratio during a working memory task assayed by fMRI (functional magnetic resonance imaging) in Val homozygotes but decreases signal-to-noise in Met homozygotes, consistent with an inverted U-shaped dose–response curve (Mattay et al., 2003).

### DA AND GAMMA OSCILLATIONS

While both DA and network oscillations have long been implicated in cognitive function, the interactions between DA and neural network activity in normal and pathological conditions have remained poorly understood. From a historical perspective this is not entirely surprising, given that DA receptor-targeting first-generation and atypical antipsychotics have proven mostly ineffective in improving cognitive deficits in SCZ patients. However, the need for a better understanding of DA effects on neural oscillations has been acknowledged (Whittington et al., 2011; Gandal et al., 2012), and a number of clinical and preclinical studies have begun to explore these relationships. While there is as yet no unified view of the effects of DA on neural networks owing at least in part to methodological and conceptual differences (*in vitro* vs. *in vivo* recordings, knock-out models vs. acute treatments, general DA signaling vs. selective modulation of distinct DA receptor subtypes), recent studies point to an important role of DA in network dynamics. Available data from human EEG studies suggests that DA increases the power of neural oscillations in the PFC, but has little effect on their induction or frequency. Carriers of polymorphisms in the DA transporter gene *DAT1* that prolong event-related increases in DA, show an increase in evoked gamma power in response to target stimuli (Demiralp et al., 2007). In healthy subjects, the first-generation antipsychotic haloperidol reduces gamma power in response to attended stimuli (Ahveninen et al., 2000), although it is unclear to what extent this effect is attributable to D2-type receptor inhibition or blockade of other haloperidol targets such as NMDA receptors.

Dopamine has been suggested to affect cognitive processes by modulating signal-to-noise ratios in PFC microcircuits (Winterer and Weinberger, 2004). Supporting indirect evidence comes from studies on working memory performance in which D1-type DA receptors optimize signal-to-noise ratio in prefrontal neural representations of non-human primates (Goldman-Rakic, 1995; Williams and Goldman-Rakic, 1995; Vijayraghavan et al., 2007). Interestingly, carriers of the *DRD4.7* allele, a subsensitive variant of the D4R (see Identification of the D4R), exhibit similar increases in auditory cortex gamma power to both standard and target stimuli in an auditory target detection paradigm, suggestive of a reduced signal-to-noise ratio (Demiralp et al., 2007).

Genetic mouse models and acute pharmacological treatments have been employed to study the effects of altered DA transmission on gamma and theta oscillations in the hippocampus and PFC. Hyperdopaminergic *DAT1* knock-out mice show increased hippocampal (but not cortical) gamma power, and enhanced gamma phase synchrony between the hippocampus and the PFC during novelty-induced exploration (Dzirasa et al., 2006, 2009). However, acute elevations of DA levels by amphetamine, apomorphine, or methamphetamine treatments had no significant effect on baseline or auditory-evoked gamma in the rodent cortex and hippocampus (Ma and Leung, 2000; Pinault, 2008; Ehrlichman et al., 2009).

Electrophysiological studies in which gamma oscillations were induced electrically or pharmacologically in rodent brain slices have yielded varying results regarding the involvement of the DA system. Ketamine-induced gamma oscillation power in the rat PFC was reduced by clozapine and haloperidol (Jones et al., 2012). Likewise, in hippocampal area CA1, DA increased the power and duration of stimulation-induced gamma oscillations (Wójtowicz et al., 2009) but decreased the power and duration of carbachol-induced oscillations (Weiss et al., 2003). However, kainate-induced gamma oscillations were found to either be decreased in CA1 and CA3 (Wójtowicz et al., 2009) or unchanged in CA3 (Andersson et al., 2012b). These variances might be partly explained by differences in the mechanisms underlying each type of oscillation.

Pharmacological analysis of the involvement of different types of DA receptors in pharmacologically induced *in vitro* gamma oscillations has revealed an unexpected wealth of targets and effects. The selective D4R agonist PD168077 augmented gamma oscillation power in CA3 (Andersson et al., 2012b); this finding will be discussed in more detail in Section “D4R and Gamma Oscillations.” Conversely, clozapine, an atypical antipsychotic with moderate selectivity for D4R and 5-HT_3_ receptors, and haloperidol reduced the amplitude and increased the bandwidth of acetylcholine-induced gamma oscillations in CA3. The effects of clozapine were attributed to inhibition of 5-HT_3_ serotonergic receptors based on the ability of the 5-HT_3_ agonist *m*-chlorophenylbiguanide to augment gamma oscillations; the same study also reported that direct stimulation of the D3R by the preferentially selective agonist PD128907 inhibited gamma oscillations (Schulz et al., 2012). Clozapine also suppressed spontaneous synchronized pyramidal network activity in layer V of the PFC (Gao, 2007). Lastly, activation of D1-type DA receptors decreased carbachol-induced gamma oscillation power (Weiss et al., 2003). Taken together, the interpretation of DA effects on oscillations are complex and likely depend on multiple factors including the brain area investigated, mechanisms of gamma oscillation induction and DA receptor pharmacology.

There is also evidence for an involvement of DA in theta oscillations. Theta frequencies frequently bind distributed networks oscillating at gamma frequency and predominate in the hippocampus during active behavior. Depleting DA in the hippocampus decreases theta activity (Nakagawa et al., 2000), while injecting DA or apomorphine into the medial septum increases hippocampal theta oscillations (Miura et al., 1987). Moreover, direct infusion of DA into the PFC of anesthetized rats increases theta oscillation coherence between the PFC and the hippocampus (Benchenane et al., 2010).

### MODULATION OF FS INTERNEURON PROPERTIES BY DA

Three key determinants of the power and frequency of gamma oscillations are (1) the magnitude and (2) kinetics of synaptic inhibition between interneurons, and (3) the driving excitatory current onto interneurons (Traub et al., 1996). Non-selective stimulation of DA receptors by exogenous DA appears not to change the electrical coupling of interneurons through gap junctions (Towers and Hestrin, 2008). However, selective activation of D1-type or D4 receptors reduces coupling (Hampson et al., 1992; Onn and Grace, 1994; Li et al., 2013) while activation of D2-type receptors increases coupling (Onn and Grace, 1994), suggesting opposing effects of different DA receptor types. Moreover, DA acting through D1-type receptors decreases the amplitude of inhibitory postsynaptic currents (IPSCs) in FS interneurons (Towers and Hestrin, 2008). DA also changes the intrinsic properties of FS interneurons by increasing their evoked firing rate *in vivo* (Tseng et al., 2006), and *in vitro* (Zhou and Hablitz, 1999; Gorelova et al., 2002; Gao et al., 2003; Kröner et al., 2007; Trantham-Davidson et al., 2008; but see Tierney et al., 2008).

By simultaneously reducing spontaneous firing and increasing evoked firing in PV interneurons, DA augments temporal precision and thereby sharpens the spike timing of FS interneurons (Gao et al., 2003; Tierney et al., 2008). Furthermore, D1-type and D2-type receptors increase the amplitude and kinetics of the h-current in layer I PFC interneurons (Wu and Hablitz, 2005). Because blockade of the h-current, an inwardly rectifying hyperpolarization-activated non-selective cation current, causes intrinsic changes in the frequency and power of many cortical oscillation frequencies (Kramer et al., 2008), it seems plausible that increases in the h-current also affect oscillations. Likewise, DA activation reduces potassium currents, thereby increasing the probability of repetitive firing, and potentially changing oscillatory patterns (Gorelova et al., 2002; Schreiber et al., 2004). Computational modeling suggests that D4Rs may modify potassium currents to increase gamma power (Kuznetsova and Deth, 2008), although experimental verification is still pending. We will discuss D4R effects on synaptic and intrinsic properties and on gamma oscillations in more detail in Sections “D4R Signaling Partnerships” and “D4R and Gamma Oscillations.”

### ROLE OF DA IN THE MATURATION OF FS INTERNEURONS DURING ADOLESCENCE

Prefrontal cortex circuits finish maturing during adolescence and early adulthood, a period of high vulnerability to first psychotic episode in persons with SCZ. Maturation involves concomitant changes in responses to DA within FS interneurons, DA receptor composition in FS interneurons, and gamma-band activity during cognitive performance. From childhood to early adolescence, gamma frequency oscillations dominate most cortical regions, and their power correlates with increases in cognitive performance. Strikingly, gamma oscillations ebb during adolescence then increase dramatically in early adulthood (Uhlhaas et al., 2009). At the end of this period, DA exerts more control over excitation/inhibition balance and thereby aids in the selection of adequate behavioral responses (O’Donnell, 2010).

Dopamine receptor expression and signaling change with age in rodents. D1Rs, D2Rs, and D4Rs increase in density in the PFC through adolescence and then show a reduction in adult animals (Zhang et al., 2004; Brenhouse et al., 2008; Naneix et al., 2012; but see Tarazi and Baldessarini, 2000). Furthermore, pharmacological blockade of D1-type receptors increases the intrinsic excitability of FS interneurons in slices from both adult (PD > 50) and juvenile (PD < 35) rats, while the D2-type receptor agonism increases FS interneuron excitability in post- but not pre-pubertal slices (Tseng and O’Donnell, 2007), thereby enhancing DA’s overall effect on intrinsic FS interneuron excitability. Similarly, in slices from pre-pubertal rats, co-administration of a D1-type agonist and NMDA excites pyramidal neurons for only tens of milliseconds, but in adults it creates plateau depolarizations lasting several hundred of milliseconds (Tseng and O’Donnell, 2005). Overall, DA receptor agonists exert more precise control over both excitatory and inhibitory neurons in adult animals, suggesting that DA may modulate oscillatory network behavior more strongly in adults.

Mature FS interneurons have faster membrane oscillations and spikes, less adaptation, and less NMDA contribution (Okaty et al., 2009; Belforte et al., 2010; Wang and Gao, 2010; Goldberg et al., 2011; Rotaru et al., 2011), and DA innervation and signaling might play a role in the maturation of FS interneurons. Treatment with DA or coculture with mesencephalic slices, which increases available DA, accelerates PV interneuron maturation in rat organotypic slices from the frontorbital cortex and increases the density of PV-positive cells in deep cortical layers (Porter et al., 1999; Ross and Porter, 2002). Moreover, elevation of prenatal DA levels by intrauterine cocaine exposure increases the ramification of PV interneurons in the anterior cingulate, a cortical area receiving dense dopaminergic innervation, but not the primary visual cortex, an area that receives little dopaminergic innervation (Wang et al., 1995). Conversely, 6-hydroxydopamine lesions in the medial forebrain bundle cause a reduction of the density of PV-expressing cells in the zona incerta of the diencephalon without reducing the total number of cells, suggesting that DA is required for the maintenance of a mature FS interneuron phenotype (Heise and Mitrofanis, 2005). These findings are consistent with the notion that the DA system is a critical modulator of GABAergic interneurons during postnatal maturation of cortical connectivity (O’Donnell, 2010) and that persons with SCZ exhibit reduced PV and GAD67 immunoreactivity (Lewis et al., 2011).

## PROPERTIES OF THE D4 DOPAMINE RECEPTOR

### IDENTIFICATION OF THE D4R

Following the cloning of the genes and cDNAs for the prototypical D2R and D1R, the gene for the human D4R (gene symbol: *DRD4*) was isolated in 1991 by homology cloning in an effort to identify additional D2-related receptors (Van Tol et al., 1991). Similar to the other D2-type receptors, it contains short amino-terminal extracellular and carboxyl-terminal intracellular tail domains, and a fairly long third-intracellular loop. DA has the highest affinity for D4R among all DA receptors (Rondou et al., 2010). Moreover, the other catecholamines epinephrine and norepinephrine bind to and activate the D4R with submicromolar affinities (Lanau et al., 1997). An outstanding feature of the human D4R is its highly polymorphic nature, owing in large part to the presence of a 48-bp variable number tandem repeat in exon III (Van Tol et al., 1992), with four and seven repeats being the most common variants overall but with substantial regional and ethnic differences in frequencies (Chang et al., 1996). By contrast, most non-primate mammal *DRD4* orthologs harbor only two repeats. The repeat encodes a proline-rich sequence located in the third intracellular loop. It conforms to a SH3 (Src Homology 3 Domain)-binding motif and has been implicated in receptor surface expression and interactions with adapter proteins such as Nck and Grb2 (Oldenhof et al., 1998). Using the amplitude of G protein-induced inwardly rectifying (GIRK) potassium currents to determine receptor activation in a heterologous *Xenopus* oocyte expression system, DA was shown to be more potent at the D4.2 and D4.7 receptor variants than the D4.4 receptor variant (Wedemeyer et al., 2007). Functional differences between receptor variants were also reported for clozapine binding, stimulation of GTPγS binding and coupling to adenylyl cyclase (Van Tol et al., 1992; Asghari et al., 1995; Jovanovic et al., 1999; Czermak et al., 2006). Numerous studies have sought to correlate polymorphic *DRD4* variants to psychiatric disorders, including association of the D4.7 variant with increased risk for ADHD (see Pharmacological Properties and Involvement in Psychiatric Disorders), and with the personality trait of novelty seeking (Ebstein et al., 1996), although some failed to replicate the original findings (for example, Hawi et al., 2000). Of particular interest to the topic of this review, D4.7 has been linked with altered cortical auditory-evoked and induced gamma-band responses, thereby implicating polymorphic D4R variants in neural oscillations (Demiralp et al., 2007; see Pharmacological Properties and Involvement in Psychiatric Disorders).

### PHARMACOLOGICAL PROPERTIES AND INVOLVEMENT IN PSYCHIATRIC DISORDERS

Initial pharmacological analyses indicated that clozapine has a higher affinity for the D4R than D2R, therefore D4R was proposed to constitute the main pharmacological target of this efficacious atypical antipsychotic (Van Tol et al., 1991). In contrast to typical or first-generation antipsychotics, extrapyramidal effects are observed to a lesser extent, or not at all, in patients treated with clozapine. While these findings generated excitement early on, subsequent studies argued against the possible utility of D4R-targeting drugs to treat the positive symptoms of SCZ. Most importantly, novel, more selective D4R antagonists such as L-745,870 and sonepiprazole were largely ineffective as neuroleptics in clinical trials (Bristow et al., 1997; Kramer et al., 1997; Corrigan et al., 2004). Consistent with this, clozapine is known to target numerous other receptor systems with low nanomolar affinities, including serotonergic (5-HT_2_), muscarinic, and β-adrenergic receptors (Baldessarini and Frankenburg, 1991), and it was concluded that its efficacy as a neuroleptic reflected a much more complex multi-target pharmacology with a significant contribution from serotonergic receptors (Meltzer and Huang, 2008). These findings, taken together with genetic association studies that largely failed to identify *DRD4* polymorphic variants as risk factors (Jonsson et al., 2003), and the inability to reproduce earlier radiolabel binding studies that suggested elevated D4R expression in SCZ subjects (Seeman et al., 1993), dampened enthusiasm to pursue the D4R as a promising antipsychotic drug target for SCZ (Tarazi et al., 2004).

However, the D4R soon re-emerged as a genetic risk factor for ADHD, a heterogeneous but highly heritable syndrome characterized by a variable combination of persistent, pervasive and developmentally inappropriate levels of inattention, hyperactivity and impulsiveness that typically lead to poor academic performance (American Psychiatric Association, 2000). Perturbations of catecholaminergic pathways are a leading hypothesis in ADHD, and dopaminergic drugs such as methylphenidate are clinically efficacious. Consistent with this, numerous ADHD candidate genes including *DRD4*, *DAT1*, *COMT*, *MAOA*, and *DBH* are integral parts of the catecholaminergic neurotransmission system. Both case–control and family-based association studies reported increased transmission of the polymorphic *DRD4.7* variant (for example, LaHoste et al., 1996; Rowe et al., 1998; Smalley et al., 1998; Swanson et al., 1998) and other polymorphisms (Barr et al., 2000; Arcos-Burgos et al., 2004) in children diagnosed with the syndrome. Subsequent meta-analyses supported a linkage between the *DRD4.7* allele and ADHD (for example, Faraone et al., 1999; Maher et al., 2002).

#### Cognitive effects of acute D4R activation

Although D4R antagonists proved ineffective as antipsychotics, D4R-targeting drugs have been examined in cognitive tasks in monkeys and rodents. For the most part, D4R agonists increase working memory performance and fear acquisition according to an inverted U-shaped dose response curve (Bernaerts and Tirelli, 2003; Browman et al., 2005; Woolley et al., 2008; but see Nayak and Cassaday, 2003); interestingly, D4R antagonists have paradoxical promnesic effects at low doses (Zhang et al., 2004). Specifically, low doses of D4R antagonists reverse working memory deficits induced by stress or chronic PCP administration in monkeys (Jentsch and Roth, 1999; Arnsten et al., 2000). In rats, D4R antagonists increase DA release in the PFC (Broderick and Piercey, 1998), possibly explaining these promnesic effects according to the inverted U-shaped dose–response curve for DA and cognition. Further supporting this idea, rats with low baseline memory performance showed the greatest improvements in response to low doses of the D4R antagonist L-745,870 and impairments at high doses, while rats with high baseline memory performance were impaired by administration of L-745,870 at every concentration (Zhang et al., 2004). In the rat, intra-medial PFC (mPFC) injection of L-741,741, another highly selective D4R inhibitor, blocks the acquisition (but not expression) of fear conditioning and the encoding of emotional memory by mPFC neurons (Laviolette et al., 2005). Conversely, medial PFC injection of the D4R agonist PD168077 increases the salience of sub-threshold foot shocks while it blocks the acquisition of fear conditioning in response to supra-threshold foot shocks (Lauzon et al., 2009; Tye et al., 2009). Additionally, the D4R agonists A-412997 and PD168077 enhance memory for aversive stimuli according to an inverted U-shaped curve (Browman et al., 2005), but have also shown linear increases in working memory performance in a similar procedure and a novel object recognition paradigm (Bernaerts and Tirelli, 2003; Woolley et al., 2008). Taken together, these results suggest that the D4R mediates memory consolidation of both normal and emotionally salient experiences, and that the intensity of the stimulus interacts with DA signaling through D4Rs.

### EXPRESSION IN FS PARVALBUMIN INTERNEURONS

A number of studies have found D4R mRNA and protein in PV interneurons of both the PFC and the hippocampus. Expression in these neurons was first observed in the monkey by double immunohistology using a carefully characterized polyclonal antibody against the extracellular amino-terminus of the receptor (Mrzljak et al., 1996). Immunoreactive cells were found in PFC layers III–V and in hippocampal area CA1. In both areas, strongly labeled PV interneurons were accompanied by lightly labeled pyramidal neurons. This study also found intense D4R immunoreactivity in the rodent globus pallidus (homologous to the primate external globus pallidus, or GPe), that is made up entirely of GABAergic projection neurons. Evidence for D4R expression in PV interneurons also comes from a single-cell RT-PCR study of acutely isolated rat PFC neurons (Vysokanov et al., 1998). Moreover, reporter gene expression was detected in PFC pyramidal neurons and interneurons, some of them expressing PV, in mice harboring a BAC transgene in which the green fluorescent protein was expressed under the transcriptional control of the *Drd4* locus (Noain et al., 2006). Two groups additionally investigated D4R expression in GABAergic interneurons using double-*in situ* hybridization for D4R and GAD67, and identified co-expressing cells in layer V of the monkey PFC (de Almeida et al., 2008) and in the mouse hippocampus (Andersson et al., 2012b). The latter study also employed double-immunohistochemistry using an antibody raised against the extracellular amino-terminus of the rat D4R (Ariano et al., 1997) and found that the majority (71%) of D4R-expressing neurons in areas CA1 and CA3 co-express PV and that conversely 21% of PV interneurons co-express D4R. In aggregate, what emerges from all these findings is the notion that in the PFC, both local GABAergic interneurons and pyramidal neurons express D4R transcript and protein, while in the hippocampus the evidence favors D4R expression largely in GABAergic interneurons.

### BEHAVIORAL PHENOTYPES OF MICE LACKING D4R FUNCTION

#### Locomotor effects

An initial behavioral analysis of mixed-background (C57Bl/6J × 129) *Drd4^-/-^* mice revealed locomotor hypoactivity and increased sensitivity to locomotor-stimulating effects of acute ethanol, cocaine, and methamphetamine injections, indicative of deficits in nigrostriatal function (Rubinstein et al., 1997). Amphetamine hypersensitivity was confirmed in a subsequent study using *Drd4^-/-^* mice congenic on the C57Bl/6J background, and expanded to also include altered behavioral sensitization to repeated amphetamine injections (Kruzich et al., 2004). At the neurochemical level, *Drd4^-/-^* mice exhibit altered striatal DA metabolism (Rubinstein et al., 1997), and lower baseline and KCl-evoked extracellular striatal DA levels (Thomas et al., 2007). In contrast, DA synthesis and turnover in the PFC of mutant mice is not significantly different (Rubinstein et al., 2001). Evidence in favor of a role of the D4R in regulating motor activity also comes from studies reporting that D4R antagonism restores normal locomotor activity in periadolescent rats rendered transiently hyperactive by neonatal 6-hydroxydopamine lesions (Zhang et al., 2001). These lesions reduce dopaminergic projections to the forebrain and serve as a neurodevelopmental model of ADHD with good face validity, reproducing a number of its core symptoms. Strikingly, 6-hydroxydopamine-mediated hyperactivity is absent in *Drd4^-/-^* mice (Avale et al., 2004). Although these findings strongly suggest a role for the D4R in mediating behavioral responses to perturbed DA function in the striatum, it remains unclear if these effects originate, as suggested, in a hyperexcitable PFC or elsewhere, and what molecular and cellular D4R-dependent processes underlie them.

#### Cognitive effects

The involvement of the D4R in cognitive functions, in particular avoidance behavior and emotional learning, has been analyzed in both mutant mice as well as in acute pharmacological paradigms (see also Cognitive Effects of Acute D4R Activation). Dulawa et al. (1999) found that *Drd4*^-/-^ mice exhibited less novelty-seeking behavior compared to wild-type controls without displaying changed anxiety. While this report concluded that D4R deficiency did not affect avoidance behavior, a different study found that *Drd4*^-/-^ mice exhibited heightened anxiety in the elevated plus maze and light/dark preference exploration tests that was ameliorated by ethanol and the benzodiazepine midazolam (Falzone et al., 2002). The fact that these anxiolytic drugs work by increasing GABAergic transmission, taken together with prominent D4R expression in interneurons in the PFC, striatal DA dysregulation and increased excitability of PFC neurons from mutant mice (Rubinstein et al., 2001), was interpreted as indicative of altered PFC control of striatal DA release in *Drd4^-/-^* mice. On the other hand, acute pharmacological inhibition of D4R in the rat mPFC by L-745,870 injection was shown to be anxiolytic in the elevated plus maze and shock-probe burial test (Shah et al., 2004). Taken together, these findings suggest that the D4R is involved in emotional learning, but that the effects of receptor interference depend on the experimental approach utilized (acute/local/pharmacological vs. chronic/global/genetic). Consistent with this notion, studies have found secondary changes in *Drd4^-/-^* mice, including upregulation of D1- and NMDA receptor expression and increased striatal glutamate that might reflect compensatory responses to a lack of D4R function in mutant mice (Gan et al., 2004; Thomas et al., 2009).

Consistent with the notion of altered PFC function in subjects with SCZ, micro-PET (positron emission tomography) imaging experiments in *Drd4^-/-^* mice indicate reduced baseline PFC glucose metabolism (Michaelides et al., 2010). Moreover, methylphenidate decreases PFC glucose metabolism in normal mice, but increases it in mutants (Michaelides et al., 2010). It is unclear to what extent these metabolic changes are directly linked to the lack of D4R function in these mice, or to compensatory changes aforementioned. Interestingly, while *Drd4* homozygous mice are normal in behavioral tests of attention and impulsivity (Helms et al., 2008; Young et al., 2011), *Drd4* heterozygous mice exhibit less response inhibition in the five choice – continuous performance task (Young et al., 2011). This observation is consistent with a lack of secondary neurochemical changes in *Drd4*^+/-^ mice (Thomas et al., 2007) and might therefore represent a true hypomorphic D4R phenotype that is not occluded by compensatory neural adaptations observed in the full mutants.

### SYNAPTIC EFFECTS OF D4Rs

#### Effects on cortical microcircuits

Cellular effects of D4R signaling in neurons have mostly been studied using electrophysiological recordings in brain slices, acutely dissociated neurons or long-term neuron cultures. Importantly, many of the effects discussed below were observed using high concentrations of the D4R agonist PD168077 that may also activate adrenergic α_1A_ and α_2C_ as well as serotonergic 5-HT_1A_ receptors (Moreland et al., 2005). At the local circuit level, Onn et al. (2006) found that in PFC slices with intact synaptic connections between axon collaterals and recorded neurons, D4R inhibition causes complex evoked spike discharges in pyramidal neurons, but only with intact GABAergic transmission. The authors suggest that DA signaling bi-directionally regulates PFC pyramidal neuron excitability via D1R and D4R-dependent pathways, respectively, and that D4Rs reduce pyramidal neuron excitability through their tonic activity by low ambient levels of DA. Consistent with these results, *Drd4*^-/-^ mice exhibit hyperexcitability of PFC pyramidal neurons and are more sensitive to bicuculline (Rubinstein et al., 2001). Although these findings indicate a possible role of D4Rs in promoting GABAergic transmission onto PFC pyramidal neurons, acute assessments of the effects of pharmacological D4R perturbations on GABAergic interneuron function have yielded mixed results, possibly stemming from the use of non-specific drugs (Gorelova et al., 2002; Gao, 2007). The possibility that D4R effects might vary between different cortical areas has to be considered as well, as pharmacological data from the prelimbic cortex suggest that D4Rs actually facilitate pyramidal neuron firing (Ceci et al., 1999).

#### Inhibition of ionotropic receptors

Various studies using neuronal preparations from the PFC, the hippocampus and the rodent globus pallidus have consistently found that pharmacological D4R activation decreases postsynaptic currents and surface expression of both excitatory and inhibitory ionotropic receptors (see **Tables 1** and **2**). GABA current amplitude and surface expression of GABA_A_R β_2/3_ clusters are decreased in PFC pyramidal neurons after treatment with relatively high concentrations (30 μM) of the D4R agonist PD168077 (Wang et al., 2002; Graziane et al., 2009). Agonist treatment also reduces GABA_A_ IPSCs in globus pallidus GABAergic neurons in wild-type mice but not in *Drd4*^-/-^ mice (Shin et al., 2003). However, D4R activation does not affect GABA current amplitude in hippocampal pyramidal neurons during ongoing kainate-induced oscillations (Andersson et al., 2012a). These data correlate with the expression of D4Rs on PFC pyramidal and globus pallidus GABAergic neurons (Mrzljak et al., 1996; Ariano et al., 1997), but not in hippocampal pyramidal neurons (Andersson et al., 2012b), and suggest that effects on GABAergic transmission are cell-autonomous.

**Table 1:** Postsynaptic effects of D4R activation on evoked and mini current amplitudes.

Current	Increase/decrease	Cell type recorded	Region	Reference
AMPA	↑ At low synaptic activity	Pyramidal	PFC	Yuen et al. (2010); Yuen and Yan (2011), but see Rubinstein et al. (2001); Onn et al. (2006)
AMPA	↓ At high synaptic activity	Pyramidal	PFC, hippocampus	Kwon et al. (2008); Yuen et al. (2010), Yuen and Yan (2011)
AMPA	↓	Interneuron	PFC	Yuen and Yan (2009); Yuen et al. (2010)
NMDA	↓	Pyramidal	PFC, hippocampus, lateral amygdala	Kotecha et al. (2002);Wang et al. (2003), Beazely et al. (2006); Wang et al. (2006), Martina and Bergeron (2008); Herwerth et al. (2012)
GABA	↓	Pyramidal, various others	PFC, hippocampus, septal nucleus, thalamus, globus pallidus, subthalamic nucleus, substantia nigra	Wang et al. (2002); Shin et al. (2003), Florán et al. (2004a, b), Asaumi et al. (2006), Acosta-Garcia et al. (2009), Graziane et al. (2009); Gasca-Martinez et al. (2010), Govindaiah et al. (2010); Andersson et al. (2012a)
Voltage-gated calcium channels	↓	Pyramidal, granule cells	PFC, cerebellum	Mei et al. (1995);Wang et al. (2006)
Potassium channel: inward rectifying	↑		Cultured neurons	Werner et al. (1996); Pillai et al. (1998), Lavine et al. (2002); Wedemeyer et al. (2007)
Potassium channel: outward current	↓	Fast-spiking interneurons	Hippocampus	Andersson et al. (2012a)

**Table 2:** Presynaptic effects of D4R activation (frequency).

Current	Increase/decrease	Cell type recorded	Region	Reference
Glutamate	↓	Pyramidal	PFC	Rubinstein et al. (2001)
Glutamate	No change	Interneuron	PFC, hippocampus	Yuen and Yan (2009); Andersson et al. (2012a)
GABA	↓	Various	Hypothalamus, septal nucleus, thalamus	Azdad et al. (2003); Baimoukhametova et al. (2004), Asaumi et al. (2006); Gasca-Martinez et al. (2010)

D4R stimulation also reduces NMDA and AMPA receptor currents. NMDA receptor currents and surface expression decrease in acutely isolated PFC and hippocampal pyramidal neurons in response to agonist treatment (Kotecha et al., 2002; Wang et al., 2003; Beazely et al., 2006), although the downstream signaling pathways may differ between the PFC and hippocampus (see D4R Signaling Partnerships). Similar effects were reported in projection neurons of the lateral amygdala (Martina and Bergeron, 2008), and in hippocampal area CA1 where NMDA receptor-dependent induction of long-term potentiation (LTP) induction in *stratum oriens* was inhibited by D4R activation (Herwerth et al., 2012). Intriguingly, in PFC slices from animals treated with the NMDA receptor antagonist PCP, a psychotomimetic drug that in humans can elicit effects remarkably similar to the symptomology of SCZ (Jentsch and Roth, 1999), D4R activation no longer inhibits NMDA receptor currents, although the cellular mechanisms underlying this effect are not known (Wang et al., 2006).

In pyramidal neurons of the PFC, but not the hippocampus, acute D4R activation by low concentrations (100 nM) of PD168077 was also shown to reduce baseline AMPA receptor currents and surface expression (Kwon et al., 2008; Yuen et al., 2010; Andersson et al., 2012a; but see Rubinstein et al., 2001; Gu et al., 2006). High concentrations of PD168077 also reduce baseline AMPA receptor currents and surface expression in PFC interneurons (Graziane et al., 2009). Furthermore, recent data suggest that D4R effects on AMPA receptors may at least in part be state-dependent. In cultured hippocampal neurons expressing the D4R, agonist treatment has no effect on baseline AMPA receptor surface expression but internalizes GluA1-containing AMPA receptors following a chemical form of LTP (Kwon et al., 2008). In PFC pyramidal cells, the D4R agonist PD168077 decreases AMPA currents when slices are pretreated with bicuculline to increase overall activity by inhibiting GABA_A_ receptors but increases AMPA currents in slices pretreated with tetrodotoxin (TTX) to reduce overall activity by inhibiting sodium channels (Yuen et al., 2010; Yuen and Yan, 2011). D4Rs also reverse early LTP at Schaeffer collateral-to-CA1 glutamatergic synapses by reversing AMPA receptor-mediated excitatory postsynaptic currents back to pre-LTP levels (Kwon et al., 2008). However, in these cases it is not known if this represents a cell-autonomous or a circuit-based effect. Taken together, with the observation that *Drd4^-/-^* mice have dramatic increases in glutamatergic signaling (Rubinstein et al., 2001), these data suggest that the D4R is involved in homeostatic processes that serve to maintain overall glutamatergic excitability within a physiological range.

#### Effects on ion channels

D4R activation decreases voltage-gated calcium channel currents in acute PFC slices and cultured cerebellar granule cells (Mei et al., 1995; Wang et al., 2006). Heterologously expressed D4Rs also couple to G protein inwardly rectifying potassium channels in *Xenopus* oocytes (Werner et al., 1996; Wedemeyer et al., 2007) through a Gβγ-dependent mechanism (Pillai et al., 1998). In principle, both activities are well suited to reduce neurotransmitter release from presynaptic nerve terminals. Consistent with this notion, the D4R agonist PD168077 reduces mIPSC frequency onto layer V pyramidal neurons, indicative of reduced GABA release from local interneurons (Gao, 2007). Moreover, D4Rs modulate transmitter release in various midbrain regions receiving GABAergic projections from the globus pallidus (Baimoukhametova et al., 2004; Florán et al., 2004a, 2004b; Asaumi et al., 2006; Acosta-Garcia et al., 2009; Gasca-Martinez et al., 2010). As mentioned earlier, globus pallidus GABAergic neurons express high levels of D4R (Mrzljak et al., 1996). Ablation of globus pallidus neurons by kainate injection prevents the inhibitory effects of PD168077 in the thalamic reticular nucleus and the substantia nigra (Acosta-Garcia et al., 2009; Gasca-Martinez et al., 2010). Effects on presynaptic calcium currents may also contribute to reduced glutamate release (Rubinstein et al., 2001; Romo-Parra et al., 2005; Yuen et al., 2010).

### D4R SIGNALING PARTNERSHIPS

Dopamine receptors were initially classified according to their ability to positively or negatively couple to adenylyl cyclase to promote the production of the intracellular second messenger cAMP. Additionally, D2-type receptors mediate many of their physiological functions via liberation of Gβγ subunits and subsequent modulation of effectors such ion channels and receptors either by direct binding or by activation of intracellular signaling pathways such as phospholipase C and mitogen-activated protein (MAP) kinase. For general reviews on DA signaling pathways downstream of heterodimeric G proteins the reader is referred to a number of excellent earlier reviews on the subject (Missale et al., 1998; Greengard et al., 1999; Gainetdinov et al., 2004; Beaulieu and Gainetdinov, 2011). In addition, D4Rs have been implicated in a non-canonical signaling process unique among DA receptors that involves the stimulation of inherent methyl transferase activity to modulate phospholipid methylation in response to agonist treatment (Sharma et al., 1999).

In the current review, we will focus on signaling partnerships of the D4R with other G protein-coupled receptors (GPCRs) and with receptor tyrosine kinases (RTK) in the regulation of neuronal function, as many of the described D4R effects in the central nervous system are tightly linked to the functional association with other receptor systems. These synergistic partnerships encompass both direct and functional interactions between receptors.

#### Partnerships with other G protein-coupled receptors

It is now widely accepted that DA receptors and other GPCRs can form homomeric and heteromeric structures, and that these multimeric aggregates can affect both ligand binding as well as signaling characteristics of their constituent subunits (Ferre et al., 2007, 2009). Recent studies have shown that the D4R heteromerizes with the both the long (D2L) and the short (D2S) forms of the D2R, alternative splice variants that differ in their third intracellular loop sequence (Borroto-Escuela et al., 2011; Gonzalez et al., 2012b). Furthermore, neurochemical evidence suggests that interactions between D4 and D2S receptors mediate DA modulation of neurotransmitter release from corticostriatal glutamatergic projections where these receptors are co-expressed (Gonzalez et al., 2012b). Intriguingly, heteromerization with D2S receptors was observed with D4R variants harboring two or four, but not seven tandem repeats, and engineered mice with the human 7R version knocked into the mouse *Drd4* locus failed to show increased glutamate release in the striatum in response to co-activation of D2Rs and D4Rs (Gonzalez et al., 2012b). These findings are especially relevant in light of the implication of the 7R allele of the human *DRD4* gene in psychiatric disorders. A second interaction was recently reported between the D4R and α_1B_ or β1 adrenergic receptors in the pineal gland where these receptors synergize to regulate circadian melatonin synthesis (Gonzalez et al., 2012a).

#### Partnerships with receptor tyrosine kinases

Functional interactions between GPCRs and RTKs have been known for many years (Daub et al., 1996). These interactions are typically functional, rather than physical, and mostly manifest as transactivation of a RTK by a GPCR (Ferguson, 2003). An early example of such transactivation involves the regulation of NMDA receptors by D4Rs (see Inhibition of Ionotropic Receptors). In PFC neurons, activation of D4R signaling downregulates NMDA receptor-mediated synaptic currents and surface expression via protein kinase A inhibition and activation of protein phosphatase 1, effects typically attributed to canonical inhibitory G protein signaling (Wang et al., 2003). However, in hippocampal neurons, D4R activation downregulates NMDA receptors via transactivation of the platelet-derived growth factor receptor (PDGFR) and downstream activation of phospholipase C/inositol triphosphate (IP3)/Ca^2^^+^ signaling (Kotecha et al., 2002). Interestingly, downregulation of NMDA receptors in the PFC in response to PDGFR transactivation by DA is mediated by D2Rs but not D4Rs (Beazely et al., 2006).

The D4R is also tightly linked to the acute effects of the NRG-1/ErbB4 signaling pathway in the central nervous system. Neuregulins comprise a family of secreted and membrane-bound factors characterized by the presence of an epidermal growth factor-like motif and that signal through ErbB RTKs to regulate a diverse array of developmental and acute processes in the peripheral and central nervous systems (Garratt et al., 2000; Buonanno and Fischbach, 2001; Falls, 2003). Moreover, both *NRG1* and *ERBB4* have been identified as risk genes for SCZ (Mei and Xiong, 2008). NRG-1/ErbB4 signaling inhibits the induction and reverses the early expression of LTP at CA3 to CA1 glutamatergic synapses (Huang et al., 2000; Kwon et al., 2005; Shamir et al., 2012). LTP reversal by NRG-1 critically depends on the activation of D4Rs (Kwon et al., 2008). Taken together with the finding that NRG-1 infusion in the dorsal hippocampus triggers DA release, this suggests that NRG-1/ErbB4 signaling regulates hippocampal LTP via a DA/D4R pathway (Kwon et al., 2008). However, it is not known if these receptor systems interact in the same or across different cell types, or which signaling pathways link their activation to the removal of synaptic AMPA receptors that underlie LTP reversal by NRG-1 and D4R agonists. For the following reasons, a direct effect on PV interneurons is more likely than on pyramidal neurons: (1) ErbB4 receptors are undetectable in pyramidal neurons (Vullhorst et al., 2009; Neddens et al., 2011) while both ErbB4 and D4Rs are expressed in GABAergic cells including PV interneurons (Mrzljak et al., 1996; Andersson et al., 2012b). (2) Inhibition of LTP induction and reversal of LTP are absent from mice with targeted deletions of ErbB4 in PV interneurons (Chen et al., 2010; Shamir et al., 2012); (3) Both receptors also appear to synergize in the modulation of hippocampal gamma oscillations that critically depend on PV interneurons (see D4R and Gamma Oscillations for details).

### D4R AND GAMMA OSCILLATIONS

Two lines of evidence suggested a possible role for the D4R in the regulation of hippocampal gamma oscillations. First, many PV interneurons contain D4R mRNA and protein (see Expression in FS Parvalbumin Interneurons). Second, earlier studies in acute rodent slices showed that NRG-1 signaling via ErbB4 potently augments kainate-induced gamma oscillations in hippocampal area CA3 (Fisahn et al., 2009). Taken together with the finding that NRG-1/ErbB4 effects on LTP reversal in CA1 critically depend on D4R signaling (see Partnerships with Receptor Tyrosine Kinases), it was plausible to hypothesize that NRG-1 effects on gamma oscillation power also depend on D4R signaling (see Buonanno, 2010). Indeed, the D4R antagonist L-745,870 largely blocks the potentiating effects of NRG-1 on gamma oscillations (Andersson et al., 2012b). Conversely, the D4R agonist PD168077 increases gamma power, albeit to a lesser extent than ErbB4 activation by NRG-1, suggesting that NRG-1/ErbB4 signaling engages multiple signaling systems that synergistically augment gamma oscillations. Neither PD168077 nor NRG-1 induce gamma oscillations in naïve slices, indicating that their modulatory effects impinge on ongoing oscillations (Fisahn et al., 2009; Andersson et al., 2012b). An investigation into the cellular effects of D4R signaling revealed that PD168077 enhances spike coherence and phase-coupling of action potentials relative to gamma cycle in FS interneurons, suggesting that D4R activation enhances gamma power by augmenting the synchronized inhibition of pyramidal neurons (Andersson et al., 2012a). Notably, pharmacological activation of other DA receptors, or direct application of DA, had no effect on gamma power. However, an enhancement of gamma power by DA was unmasked by simultaneous application of the D1-type receptor blocker SCH23390, indicating that D1-type DA receptors antagonize the effects of D4Rs on gamma oscillations (Andersson et al., 2012b).

Interestingly, D4R-mediated augmentation of gamma power is sensitive to pharmacological blockade of NMDA receptors by AP5 (D-2-amino-5-phosphonopentanoate), although AP5 has no effect on gamma power *per se* (Andersson et al., 2012b). Given that D4Rs internalize NMDA receptors (see Inhibition of Ionotropic Receptors), a simple explanation might be that D4R activation improves excitation-spiking coupling by removing NMDA receptors from excitatory synapses of FS interneurons to increase gamma power. Indeed, blockade of NMDA receptors increases gamma power *in vivo* (Pinault, 2008). Yet, if this were true, blocking NMDA receptors would occlude rather than inhibit the D4R effect on gamma power, and would increase gamma power even in the absence of D4R agonist. However, as mentioned earlier, this was not observed *in vitro* (Andersson et al., 2012a). At this time, the relationship between D4R signaling and NMDA receptor function in kainate-induced hippocampal oscillations is therefore unclear.

Studies exploring the role of D4Rs in gamma oscillations in the PFC are currently lacking. Considering the conserved expression of D4Rs and ErbB4 receptors in PV interneurons in the hippocampus and neocortex, it is reasonable to speculate that synergistic signaling downstream of both signaling pathways also modulates neocortical gamma rhythms. On the other hand, there are significant differences between the hippocampus and the PFC. For example, D4R activation reduces NMDA currents via PDGFR transactivation in the hippocampus and via canonical G_ai_ signaling the PFC (see Partnerships with Receptor Tyrosine Kinases). Furthermore, D4Rs in the PFC are expressed on both interneurons and pyramidal neurons, while in the hippocampus pyramidal neurons mostly do not express the receptor (see Expression in FS Parvalbumin Interneurons). However, indirect evidence already exists that D4Rs in the human cortex modulate gamma oscillations. Carriers of the 7R *DRD4* allele (*DRD4.7*) show enhanced evoked and induced gamma oscillations in the frontal and association cortices in response to auditory stimuli (Demiralp et al., 2007). This increase in evoked gamma oscillations does not discriminate between target and filler stimuli, suggesting that it is harder for subjects to distinguish between salient and distracting stimuli, a finding that is consistent with the association of the *DRD4.7* allele with ADHD and potentially cognitive function in other psychiatric disorders (see Pharmacological Properties and Involvement in Psychiatric Disorders). Importantly, the 7R variant has half the potency of more common third-loop repeat variants for inhibiting cAMP (Asghari et al., 1995) and less effectively forms heterodimers with the long and short variants of the D2R (Borroto-Escuela et al., 2011; Gonzalez et al., 2012b). If the modulation of gamma rhythms in individuals carrying the *DRD4.7* polymorphism is indeed compromised, the prediction would be that normal D4R function enhances signal-to-noise ratios by suppressing gamma rhythms in response to non-salient sensory stimuli. At least in principle, the finding that D4Rs decrease AMPA currents in interneurons in the PFC (Yuen and Yan, 2009), and that decreased excitatory drive onto PV interneurons reduces gamma oscillation power (Rotaru et al., 2011), correlates well with increases in gamma in subjects carrying the *DRD4.7* allele.

## OUTLOOK

The association of gamma oscillations with attention, working memory and other cognitive functions, and their perturbation in numerous psychiatric disorders, underscores the potential therapeutic value of targeting networks that generate and regulate gamma oscillations (see Andersson et al., 2012b). Based on work reviewed herein, we propose that a potential functional role for DA in modulating cognitive processes is through opposing effects on neuronal excitability, synaptic strength, and gamma oscillations mediated via D1- and D2-type DA receptors (see Kwon et al., 2008; Andersson et al., 2012b). Consequently, the regional and temporal dynamic changes of DA concentrations in the hippocampus and PFC could determine the D1-/D2-type receptor activity ratio that ultimately modulates gamma oscillation power and synchrony at rest (baseline) and evoked by salient stimuli; the relative activity of both receptor types could account for the inverted U-shaped dose–response relationship associated with DA effects. The D1-/D2-type receptor activity ratio is especially important for the regulation of GABAergic function in the PFC (Seamans et al., 2001; Goldman-Rakic et al., 2004; Trantham-Davidson et al., 2004; Enomoto et al., 2011), where excitatory/inhibitory balance is critical for the optimization of cognitive task performance and flexibility (Winterer and Weinberger, 2004). Because optimal signal-to-noise ratio of cortical microcircuits is important for cognitive tasks (Winterer and Weinberger, 2004), and alterations in gamma frequency oscillations are associated with psychiatric disorders (Herrmann and Demiralp, 2005; Uhlhaas and Singer, 2010), identifying modulators that improve signal-to-noise ratio by targeting cortical microcircuits could constitute a promising therapeutic approach.

We propose, based on the functional properties and expression of ErbB4 and D4Rs in GABAergic PV-expressing interneurons in the DLPFC (Mrzljak et al., 1996) and hippocampus (Andersson et al., 2012b), as well as their effects on kainate-induced gamma oscillations and plasticity (Kwon et al., 2008), that these receptors are well situated to modulate network activity that affects cognition (see Partnerships with Receptor Tyrosine Kinases, and D4R and Gamma Oscillations). Although D4R-specific antagonists were found to be ineffective as antipsychotics for the treatment of SCZ (see Pharmacological Properties and Involvement in Psychiatric Disorders), D4R-targeting drugs still hold potential as adjunct therapies to ameliorate the cognitive deficits seen in SCZ (see Andersson et al., 2012b). The fact that D4R activity can regulate working memory and other cognitive behaviors in rodent models (Zhang et al., 2004; Braszko, 2009; Young et al., 2011), and that their effects are state-dependent (Zhang et al., 2004), suggests that D4R modulators could be used to modulate D1-/D2-type receptor ratio and improve signal-to-noise ratio. Given that the cognitive responses to D4R-targeting drugs depend on emotional salience and baseline ability that may easily reflect basal DA concentration, it is plausible that people with different variants of the D4R may respond differently to these therapies. D4R agonists may prove more efficacious in some patients, while D4R antagonists may prove more efficacious in others, and oscillatory activity may help predict which therapy will be more effective.

While pharmacological targeting of D4Rs for regulating network activity and cognitive function appears to be a promising approach, as we have elaborated throughout this review, one must be mindful of the many complexities of D4R signaling when designing and interpreting meaningful experiments for future studies. These considerations include the likelihood that the cellular D4R expression pattern varies in different cortical regions, that drug effects on D4R function might be dose-dependent and that the receptor might engage in distinct signaling partnerships in different cells or subcellular compartments. The challenge will therefore be to find among the abundance of possibilities the ones that are pertinent to the regulation of local circuit activity and their potential to improve cognitive processes deficient in a number of neurological and psychiatric disorders. Perhaps treatments tailored to increase or reduce D4R activity in different patients holds promise for approaches that for the past decades have failed to improve working memory and other cognitive functions in these disorders (see Insel, 2010).

## Conflict of Interest Statement

The authors declare that the research was conducted in the absence of any commercial or financial relationships that could be construed as a potential conflict of interest.
